# Examination of Staffing Shortages at US Nursing Homes During the COVID-19 Pandemic

**DOI:** 10.1001/jamanetworkopen.2023.25993

**Published:** 2023-07-27

**Authors:** Joan F. Brazier, Fangli Geng, Amy Meehan, Elizabeth M. White, Brian E. McGarry, Renee R. Shield, David C. Grabowski, Momotazur Rahman, Christopher Santostefano, Emily A. Gadbois

**Affiliations:** 1 Department of Health Services, Policy & Practice, Brown University School of Public Health, Providence, Rhode Island; 2 Student, PhD Program in Health Policy, Harvard University, Cambridge Massachusetts; 3 Department of Health Care Policy, Harvard Medical School, Boston, Massachusetts; 4 Division of Geriatrics and Aging, Department of Medicine, University of Rochester Medical Center, Rochester, New York

## Abstract

**Importance:**

Staffing shortages have been widely reported in US nursing homes during the COVID-19 pandemic, but traditional quantitative research analyses have found mixed evidence of staffing shortfalls.

**Objective:**

To examine whether nursing home administrator perspectives can provide context for conflicting aggregate staffing reports in US nursing homes during the COVID-19 pandemic.

**Design, Setting, and Participants:**

In a qualitative study, convergent mixed-methods analysis integrating qualitative and quantitative data sets was used. Semistructured qualitative interviews were conducted between July 14, 2020, and December 16, 2021. Publicly available national Payroll Based Journal data were retrieved from January 1, 2020, to September 30, 2022, on 40 US nursing homes in 8 health care markets that varied by region and nursing home use patterns. Staffing and resident measures were derived from Payroll Based Journal data and compared with national trends for 15 436 US nursing homes. Nursing home administrators were recruited for interviews. Of the 40 administrators who consented to participate, 4 were lost to follow-up.

**Exposure:**

Four repeated, semistructured qualitative interviews with participants were conducted. Interview questions focused on the changes noted during the COVID-19 pandemic in nursing homes.

**Main Outcomes and Measures:**

Thematic description of nursing home administrator compensatory strategies to provide context for quantitative analyses on nursing home staffing levels during the COVID-19 pandemic.

**Results:**

A total of 156 interviews were completed with 40 nursing home administrators. Administrators reported experiencing staff shortages during the COVID-19 pandemic and using compensatory strategies, such as overtime, cross-training, staff-to-resident ratio adjustments, use of agency staff, and curtailing admissions, to maintain operations and comply with minimum staffing regulations. Payroll Based Journal data measures graphed from January 1, 2020, to September 30, 2022, supported administrator reports showing that study facilities had reductions in staff hours, increased use of agency staff, and decreased resident census. Findings were similar to national trends.

**Conclusions and Relevance:**

In this qualitative, convergent mixed-methods study, nursing home administrators reported the major staffing strain they experienced at their facilities and the strategies they used to offset staffing shortages. Their experiences provide context to quantitative analyses on aggregate nursing home census data. The short-term compensatory measures administrators used to comply with regulations and maintain operations may be detrimental to the long-term stability of this workforce.

## Key Points

**Question:**

Do nursing home administrator perspectives on staffing in US nursing homes during the COVID-19 pandemic provide context for conflicting staffing data reports?

**Findings:**

In this study, qualitative and quantitative data from 40 US nursing homes were integrated to assess staffing levels during the pandemic. Short-term compensatory strategies were used by administrators to comply with minimum staffing regulations and offset staffing shortages.

**Meaning:**

Findings from this study suggest that staffing shortages during the COVID-19 pandemic placed strain on nursing homes.

## Teaser

This qualitative study examines the strategies used by nursing home administrators to cope with changes in staffing during the COVID-19 pandemic.

## Introduction

The COVID-19 pandemic has put tremendous strain on the US nursing home workforce.^1,2,3^ Burnout,^4,5^ low wages,^6^ poor work conditions,^7^ and the increased burden of caring for vulnerable residents during a health crisis^3,8^ have contributed to a 13.3% decrease in nursing home sector employment since the start of the pandemic.^9^ Nursing homes currently employ 1.37 million workers (roughly 10% below projected demand)^9^ and continue to face staff shortages.^10,11^ Administrators have responded to ongoing staff shortages by increasing staff workloads,^12,13^ halting or decreasing new admissions,^14,15,16^ and offering substantial wage raises^10,17^ and other incentives to retain staff.^18^ Despite these efforts, only 2% of all nursing homes in the US reported being fully staffed in 2022.^19,20^

Although staff shortages at nursing homes have been widely reported,^12,18,19,21^ quantitative studies have found mixed evidence of staffing shortfalls. One study found no decrease in staffing levels during the early part of the pandemic after accounting for a decreased census.^22^ The Kaiser Family Foundation reported that nursing home staffing shortages coincided with COVID-19 variant surges, varied widely by state, and peaked in January 2022 at 34%.^11^ In contrast, an analysis that used detailed employee-level payroll data found staffing patterns consistent with reports of nursing homes experiencing major staffing challenges during severe COVID-19 outbreaks and for extended periods of time after the outbreak.^23^

This study conducted a qualitative assessment of nursing home administrator experiences during the pandemic and integrated qualitative findings with quantitative analysis of national payroll staffing data. The objective was to provide context to conflicting aggregated data on nursing home staffing levels during the COVID-19 pandemic.

## Methods

This convergent mixed-methods study^24,25,26^ used semistructured qualitative interviews with nursing home administrators and merged thematic results with quantitative analyses of publicly available facility-level staffing data. This project followed the Consolidated Criteria for Reporting Qualitative Research (COREQ) reporting guideline for qualitative research and was approved by the Brown University Institutional Review Board, which determined it to not be human research. Verbal consent was obtained prior to audiorecording interviews. The nursing home administrators received compensation for participation.

### Qualitative Methods

Using the Hospital Referral Region table from the Centers for Medicare & Medicaid Services Geographic Variation Public Use file, 8 health care markets were identified.^27^ Markets varied based on US region and nursing home use patterns. Using purposive sampling, 5 nursing homes that varied by 5-star rating, size, payer mix, and profit status were selected in each market.^28^ Administrators were recruited by email and telephone to participate in semistructured interviews. Interviews were repeated at 3-month intervals from July 14, 2020, to December 16, 2021, to understand the outcomes associated with COVID-19 in US nursing homes over time.

Interview protocol development and testing consisted of 3 cognitive interviews with the immediate research team and 3 pilot interviews with nursing home administrators, after which final revisions to the interview guide were made. The interview guide included open-ended questions and subsequent probes about COVID-19 at nursing homes and was used as a baseline across all 4 interviews, with modifications to add or discontinue questions as needed. Interview guides for interviews 2 and 3 included follow-up questions specific to the facility’s previous interview. A summary report detailing preliminary findings and emerging themes was sent to participants before their third interview. During the third interview, targeted questions were asked to solicit feedback on the summary report, confirm preliminary findings, and refine emerging themes. The interview guide for interview 4 was further modified to include questions designed to look back on administrator experiences over the 1-year interview time frame (eMethods in Supplement 1).

Four qualitative research team members (J.F.B., A.M., R.R.S., and E.A.G.) conducted the interviews. All were women with 5 to 35 years of experience in conducting qualitative research. They included 2 PhD-level faculty members and 2 Master’s-level research staff. The researchers did not know the interview participants before the first interview. The purpose of the research was shared with interview participants during recruitment and at the start of each interview.

Interviews were conducted virtually or by telephone depending on participant preference and lasted approximately 60 minutes. Two qualitative research team members participated in each interview: one conducted the interview while the other took detailed notes to flag questions for follow-up and record emergent themes.

Interviews were recorded, professionally transcribed verbatim, deidentified, and reviewed for accuracy. Transcripts were not shared with participants. Using modified grounded theory,^29^ an initial coding scheme was developed based on the interview guide (a priori codes) and on emerging data from interviews (de novo codes). The coding tree was adjusted iteratively, such that codes were added and refined throughout data collection and analysis. Four researchers (J.F.B., A.M., R.R.S., and E.A.G.) double-coded 102 interview transcripts in coding teams of 2. Teams rotated to ensure rigor and prevent drift in code definition understanding. Preliminary emerging themes were identified and noted in an audit trail. Once high coding agreement was reached, 54 transcripts were coded by individual researchers. Coded transcript data were entered into the qualitative software package NVivo, version 12 Plus (QSR International) to facilitate comparative analyses across themes.

Once all interview transcripts were coded, reports were generated that collected all the quotations assigned to the same code and related to an identified preliminary theme. Using the 6 steps devised by Braun and Clarke for thematic analysis,^30^ the code reports related to a theme were examined together and reanalyzed to identify quotations that were both supportive of and in contrast to the identified themes and identify additional themes. It was determined that saturation was achieved.^31^ During analysis, a comprehensive audit trail^32^ was kept to record team decisions, questions and comments, code definitions, and emerging themes.

### Quantitative Methods

Daily facility-level staffing data for January 1, 2020, to September 30, 2022, were obtained from the publicly available Centers for Medicare & Medicaid Services Long-term Care Facility Staffing Payroll Based Journal (PBJ) data.^33^ All Medicare- or Medicaid-certified nursing homes are required to submit daily staffing data, which includes hours worked by staff type and contract type (ie, agency vs direct employee) and resident census. To construct national averages for comparison, data for the 40 sample nursing homes and all 15 436 nursing homes in the US were obtained.

The PBJ data were used to construct 4 measures between January 2020 and September 2022. Measure 1: the mean total daily direct care staff including registered nurses (RNs), licensed practical nurses (LPNs), and certified nursing assistants (CNAs) in any given week; measure 2: the mean daily resident census in any given week; measure 3: direct care hours per resident-day, calculated by dividing total hours by patient census on that day^34^; and measure 4: the share of agency worker hours (rather than direct care employees) that provide temporary staffing to offset potential shortages of RNs, LPNs, and CNAs. The staffing measures provide insights into the adequacy of staffing levels and quality of care during the study period. The share of agency worker hours measure is an indicator of staffing stability and may reflect challenges in recruiting and retaining direct care employees.

Weekly means for the 4 measures for the 40 participating nursing homes and 15 436 nursing homes nationally were calculated. These averages were graphed over time to examine temporal trends in the study sample and compared with national trends for the same time period. To construct the national average for each measure, data were weighted by the size of the nursing home, as measured by the number of residents. To ensure the accuracy of our analyses, any data points with either the resident census or the total staffing reported as 0 for a facility on any given day were excluded.

### Integration of Data Sets

Qualitative and quantitative data sets were integrated to answer the question, Do nursing home administrator perspectives on staffing in US nursing homes during the COVID-19 pandemic provide context for conflicting staffing data reports? The 3 themes generated by qualitative analysis prompted the development of 4 analytic measures to statistically evaluate facility-level data for the study sample. Qualitative themes were compared with quantitative measures to assess whether administrator experiences with nursing home staffing levels were supported by facility-level data and whether administrator perspectives provided an explanation for how staffing challenges were addressed.

## Results

This mixed-methods study included 156 total interviews with 40 nursing home administrators in 8 markets across the US. Although specific demographic information was not gathered, participants were licensed nursing home administrators who self-reported a range of education levels and backgrounds, including nursing, social work, business administration, health care administration, public administration, finance, and marketing. Experience in nursing homes ranged from several months to more than 30 years. Nursing home characteristics are presented in Table 1.

**Table 1.** Facility Characteristics

Facility ID	Region	Bed count category, No.	For-profit	% Medicare category	Star rating^a^		
					Facility	Staff	RN
S1N1rep	Northeast	126-150	Yes	0-9.9	1	3	3
S1N2	Northeast	100-125	No	10-19.9	3	5	5
S1N3	Northeast	100-125	Yes	10-19.9	5	3	5
S1N4	Northeast	<100	Yes	10-19.9	2	4	4
S1N5	Northeast	<100	Yes	10-19.9	4	2	3
S2N1	Northeast	<100	No	10-19.9	4	3	3
S2N2	Northeast	100-125	Yes	10-19.9	5	3	3
S2N3	Northeast	≥151	Yes	10-19.9	1	3	3
S2N4	Northeast	≥151	No	10-19.9	3	2	1
S2N5	Northeast	100-125	No	0-9.9	2	3	3
S3N1	South	≥151	Yes	10-19.9	1	2	1
S3N2	South	100-125	No	≥30	5	5	5
S3N3	South	126-150	Yes	0-9.9	2	3	3
S3N4	South	<100	Yes	10-19.9	3	3	4
S3N5	South	<100	Yes	≥30	4	4	5
S4N1	Midwest	126-150	Yes	10-19.9	2	5	5
S4N2	Midwest	<100	No	≥30	5	5	5
S4N3	Midwest	100-125	Yes	10-19.9	1	5	5
S4N4	Midwest	≥151	No	0-9.9	3	4	5
S4N5	Midwest	≥151	No	10-19.9	4	5	5
S5N1	West	<100	Yes	10-19.9	2	1	1
S5N2	West	100-125	Yes	10-19.9	3	4	4
S5N3	West	<100	Yes	10-19.9	5	4	4
S5N4	West	126-150	Yes	10-19.9	2	3	3
S5N5	West	<100	Yes	0-9.9	4	4	3
S6N1	South	126-150	No	≥30	4	4	4
S6N2rep	South	100-125	Yes	≥30	5	3	3
S6N3	South	100-125	No	0-9.9	2	2	2
S6N4	South	100-125	Yes	10-19.9	1	3	3
S6N5	South	100-125	Yes	≥30	3	4	4
S7N1	South	<100	No	≥30	4	3	2
S7N2	South	126-150	Yes	≥30	1	1	1
S7N3	South	<100	Yes	≥30	2	1	1
S7N4	South	≥151	Yes	20-29.9	3	1	1
S7N5	South	<100	No	20-29.9	5	4	4
S8N1	West	<100	Yes	10-19.9	3	2	2
S8N2	West	≥151	Yes	10-19.9	2	4	4
S8N3	West	≥151	Yes	20-29.9	5	3	4
S8N4	West	≥151	Yes	10-19.9	4	2	2
S8N5	West	<100	Yes	≥30	2	2	2

Abbreviation: RN, registered nurse.

^a^ Star rating is a quality measure developed by the Centers for Medicare & Medicaid, with 1 being much below average quality and 5 being much above average quality.

Using modified grounded theory^29^ and thematic analysis,^30^ 3 major themes that reflect administrator perspectives on nursing home staffing from July 14, 2020, to December 16, 2021, were identified. In theme 1, administrators report on the substantial staffing shortages they experienced during the pandemic. Themes 2 and 3 present major strategies administrators used to offset immediate staffing shortages including hiring agency staff (theme 2) and operating at a reduced resident census (theme 3). Embedded within each theme are concepts that support the theme. Themes, concepts, and illustrative quotations are summarized in Table 2.

**Table 2.** Themes, Concepts, and Illustrative Quotations

Theme and concept	Representative quotation (ID, date, region, size, facility star rating^a^)
**Theme 1: Administrators report substantial staff shortages during the pandemic**	
Shortage of direct care staff	With that many staff members out [due to COVID-19 infection], we had an extreme staffing crisis. There was nobody to help. (S5N1.3, March 2021, West, <100 beds, 2 star)
	We still are [struggling with staffing shortages], and every nursing home in the city is struggling. We’re all using agency, and even agency can’t fill our open positions. (S3N2.4, August 2021, South, 100-125 beds, 5 star)
	There is a staffing shortage especially of CNAs in this county. It’s pretty bad. We have a lot of openings. So we finagle a lot to try to meet the needs of the residents and [the] need or requirement of PPD. (S6N4.1, November 2020, South, 100-125 beds, 1 star)
Compliance with minimum staffing regulations	And my first number 1 goal is to make sure that I meet these minimum staffing requirements, whatever it takes, and whatever I have to do, and if it means I have to make decisions that I ordinarily would not have made to benefit patient care, then so be it. (S1N5.3, April 2021, Northeast, <100 beds, 4 star)
	Our census, we’re budgeted for 137, and we’re at 126. But, we’re holding on purpose at 126. And the reason for that is because staffing is such a challenge....We’re still not where we want to be for our per person ratio of staff to residents. However, it’s safe, and they’re still getting good care. But we feel like if we took any more, they wouldn’t be getting that good customer service. Their care might be compromised, you know, because we don’t have enough staff. (S2N4.4, August 2021, Northeast, ≥151beds, 3 star)
	We have ratios to meet and daily averages. So there’s quite a few things we have to monitor to be in compliance when it comes to staffing. And sometimes I actually have to curtail admissions because I cannot meet our daily per person per day hours. (S6N4.3, June 2021, South, 100-125 beds, 1 star)
Strategies to manage staff shortfalls: overtime and cross-training	I continue to have staffing issues. We have 22 open FTEs with nursing positions, direct care positions. I’ll just do some quick math here, 22 times 2080, is 45 760 h divided by 365 is 125 h divided by 8-hour shifts, that’s 15.6 positions a day that I just don’t have staffed. So our overtime is obscene…I just can’t get staff. (S4N5.3, May 2021, Midwest, ≥151 beds, 4 star)
	And what we’ve opted to do is that from management all the way down we have cross-trained individuals to move into areas where they don’t normally work. But we’ve asked them to, “You know what, we need someone here.” (S6N1.1, August 2020, South, 126-150 beds, 4 star)
Strategies to manage staff shortfalls: higher staff-to-resident ratios	We usually run a 1 to 10 ratio with our CNAs. And if we’re running 3 [CNAs], then of course, we’re going to be more than 1 to 13, 1 to 14 ratio, which can be done. Some of these facilities are running 1 to 20 ratio with their CNAs. You’re hearing horror stories. But where we have nurse management that’s on the floor, they’re filling those voids, where we have that extra nurse on the weekends, we’re filling those voids. (S3N5.3, September 2021, South, <100 beds, 4 star)
	They’re tired because you got, our ratio is usually 1 to 7, 1 to 8 people. It’s much higher now. (S7N5.4, December 2021, South, <100 beds, 5 star)
**Theme 2: Agency staff were hired to offset staffing shortages**	
Need to use agency staff to meet staff/patient ratio regulations	We’re still utilizing agency, and all of that to be able to keep the building staffed according to the guidelines. (S6N4.4, September 2021, South, 100-125 beds, 1 star)
	I just hired starting mid-March a traveling CNA that will come and be a part of our team for 8 wk that will take some of the pressure off our staffing and allow us to go to that higher census level that we need to go to. You have to have CNAs in [state] based on your census, so for example if I have 7 CNAs on day shift, I can go to a census of 49 because they have 7 patients a piece. But if I want to go to 50 I have to add another CNA. (S5N3.2, March 2021, West, <100 beds, 5 star)
First time using agency staff	My previous administrator here, as well as who happens to be a regional vice president of our region, he says, “We’ve never used agency in 20 years I was there,” and I was the first one to use them. I felt bad financially because that’s obviously, you have to pay more, but at the same time, we can’t leave our staff stranded. (S8N3.4, July 2021, West, ≥151 beds, 5 star)
	This is the first time I’ve ever had to use agency….We had to use a lot of agency staff....You have people that aren’t invested into your residents that goes in your building because they don’t know them. Right? And so you don’t have that bond like you would with one of your own permanent employees or that level of dedication. So I think that is tough. (S2N4.2, February 2021, Northeast, ≥151 beds, 3 star)
High demand for agency staff	We contracted with several agencies, but there was such a high demand for agency use at the time and the companies, the long-term care companies as well as hospitals I’m sure were all outbidding how much they were going to pay for an agency nurse. (S2N2.1, October 2020, Northeast, 100-125 beds, 5 star)
	I was authorized to get agency nursing and CNAs. But the fact is, every other nursing home was going through the same thing. So, there were only so many agency nurses or CNAs who would go anywhere. (S1N1rep.1, April 2021, Northeast, 126-150 beds, 1 star)
High cost of agency staff	We’re still using agencies, we’re actually having to start using agencies for nurses. There’s still a huge nursing shortage because now hospitals in our area they’re starting to hire first-year LPNs and RNs, and usually, that’s our bread and butter. We pair with the colleges and have clinicals held here and bring in the nurses, but now that the hospitals are so short-staffed too they’ve opened up the first-years and higher wages so we’ve been losing staff to the hospitals. (S5N4.2, May 2021, West, 126-150 beds, 2 star)
	Today I probably get 1-2 agencies call me a day asking me to sign up with their agency. The problem is there’s only a limited number of CNAs in the market, so they’re all vying for the same candidates and none of them can actually produce. And none of them want to block book a CNA because if we block book at $23 an hour, now their cut is smaller, but if they let us fight each other to get up to $32 an hour now their cut’s larger. It’s not about the agencies supporting the facilities to actually be a benefit, it’s about the agencies making money. (S6N3.3, April 2021, South, 100-125 beds, 2 star)
Agency staff prioritizing monetary reward	[Staffing] was difficult at best and we depended very, very heavily on contracted staff from out of state and other parts of the state and people who, realistically, were chasing hazard pay and following the bug around all over the place. (S2N3.1, October 2020, Northeast, ≥151 beds, 1 star)
	But with COVID, [hiring nurses has] gotten worse and harder. And then here’s a staffing agency moves right in. And this nurse, instead of me paying them $20 an hour, they can get paid $30/h, go work for the staffing agencies, pick and choose when they want to work, where they want to work, and they have no commitment. They just come in, collect a paycheck, and walk out. (S7N4.2, March 2021, South, ≥151 beds, 3 star)
Challenges of agency staff impact on morale	I’ve had issues with staff morale. They’re tired because staffing is an issue. We have a lot of trouble with keeping permanent staff and have had to use agency staff and that always seems to, in the facilities I’ve been in, that seems to affect the morale. But we have put in some other hiring incentives and are starting to see a little bit of a turnaround, but that’s the biggest change with staff, not enough of them and morale has been hard to keep up still. (S7N2.3, April 2021, South, 126-150 beds, 1 star)
	I think the challenge now is most of our staffing, we are using agency for CNAs and nurses after many years of being agency-free. So I think that is the challenge. And if you don’t have a traveler, which now we do, it makes it even worse, because you don’t have any type of consistency on your schedule. (S2N2.4, July 2021, Northeast, 100-125 beds, 5 star)
Challenges of agency staff on patient care	We did use some agency on our COVID unit, but that’s worrisome too, because you’ve got really sick patients that you love, and people that may not know them, and families that may not know those staff, so we were trying to balance that and make sure that we had at least some regular staff along with agency staff in the COVID unit all the time. (S5N3.1, December 2020, West, <100 beds, 5 star)
	I’d rather pay my people triple pay than to get an agency. They’re just terrible. (S7N5.2, May 2021, South, <100 beds, 5 star)
**Theme 3: Administrators report being unable to grow their census due to continued staffing shortages**	
Efforts to grow resident census impacted by staff shortages	Yeah, the only restraint on us getting a little bit higher census is staffing issues. We’re running into staffing issues. (S5N2.4, July 2021, West, 100-125 beds, 3 star)
	Well, I think more just after the whole year. Just trying to catch up to where we used to be, and with census as well. I mean, that fluctuates how many staff we have, too. So…we’re trying to get more staff in, and still trying to build our census so they feel they have more help. They’re not so burnt out, and with the staff turnover that we have had, just trying to replace them. (S8N4.2, April 2021, West, ≥151 beds, 4 star)
Low staffing levels impacting census	It’s going to be concerning for a budgetary purpose because, with low census, we’re already crunching numbers now, having to crunch staff members and making sure that we’re just running what we need to run in order to stay afloat, maintain a positive operation. (S3N5.2, May 2021, South, <100 beds, 4 star)
	I’d say that’s the primary way that it’s [staffing shortages] affecting [my facility] is just by keeping census very low. We have facilities in the area that have shut down entire wings of their buildings. Thankfully, we have not had to go to such drastic measures. So, whereas we have had to reduce our patient census by about 10%-15% of what we normally see, many other buildings have had to reduce their patient census by 30%-50%. (S5N5.3, September 2021, West, <100 beds, 4 star)
Capping admissions due to staffing levels	Then, right around that same time [COVID outbreak], things I guess eroded as far as staffing goes, so we just have not had the staff that we’ve needed. This will probably come up later in the interview, but we had to contract with a staffing agency to provide temporary staffing and they could not keep us staffed even though we were paying roughly double the rate that we would normally pay for staff. Because of that, we haven’t been able to admit patients like we normally would, so we’ve been having to cap admissions and cap the census really at around anywhere from 105 to 109 over the last probably 30-45 d or more. (S6N2rep.2, June 2021, South, 100-125 beds, 5 star)
	The volume of admissions has reduced due to staffing. So as a result of being short staffed, we’ve not been able to admit near as many residents as we would like, so our census has dropped by about an average of 10 per day due to the staffing. So we’re having to get creative with staffing. (S6N3.2, January 2021, 100-125 beds, 3 star)

Abbreviations: CNA, certified nursing assistant; PPD, per patient day.

^a^ Star rating is a quality measure developed by the Centers for Medicare & Medicaid, with 1 being much below average quality and 5 being much above average quality.

**Figure 1.**
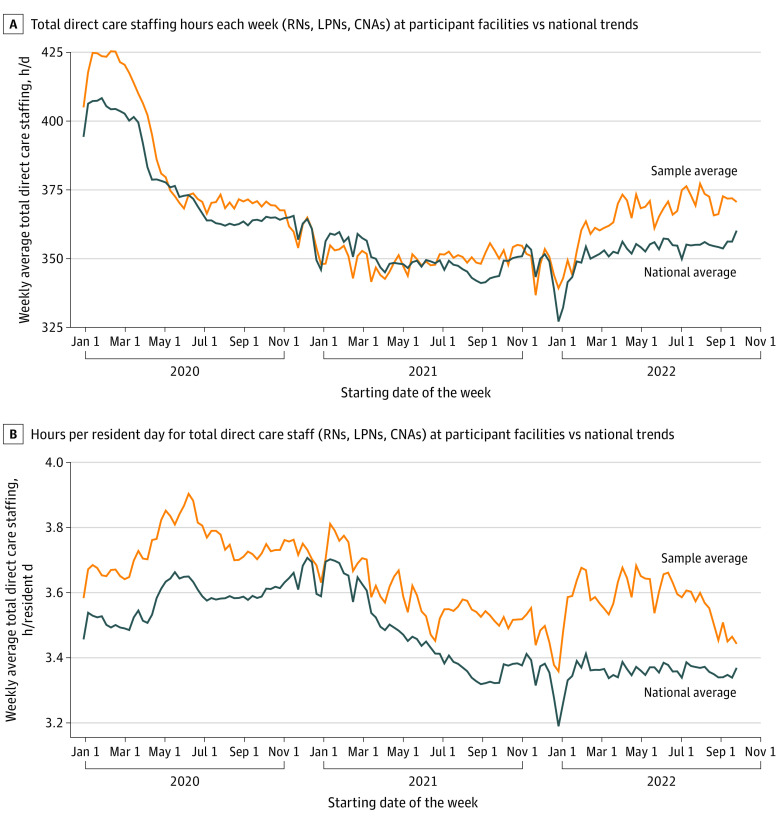
Staffing Hours and Hours per Resident-Day A, Total direct care staffing hours each week (registered nurses [RNs], licensed practical nurses [LPNs], and certified nursing assistants [CNAs]) at participant facilities and compared with national trends. B, Hours per resident-day for total direct care staff (RNs, LPNs, and CNAs) at participant facilities and compared with national trends.

**Figure 2.**
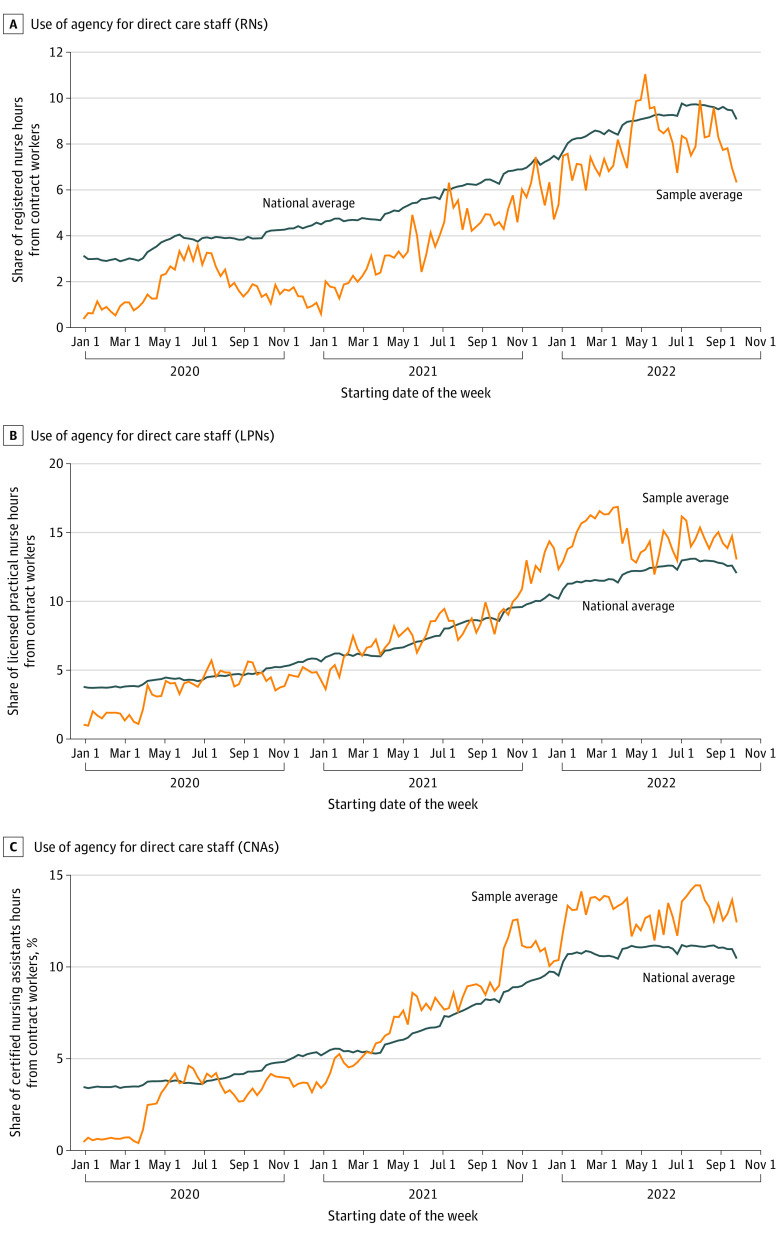
Use of Agency Staff A, Use of an agency for direct care staff including registered nurses (RNs) (A), licensed practical nurses (LPNs) (B), and certified nursing assistants (CNAs) (C).

**Figure 3.**
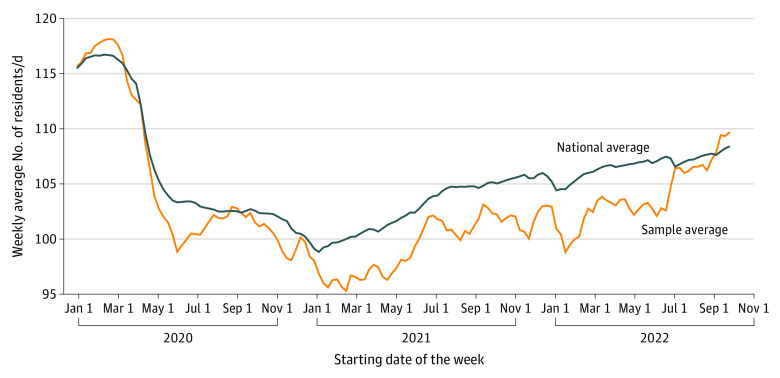
Resident Census for 40 Samples for Each Week

Quantitative analysis of facility-level staffing data assessed changes found in the study sample of nursing homes from January 1, 2020, to September 30, 2022. In measure 1, study nursing homes experienced large reductions in total direct care staff hours throughout the study period (Figure 1A). Measure 2 showed that study nursing homes experienced reductions in direct care staff hours per resident-day throughout the study period (Figure 1B). Measure 3 indicated that the census at study nursing homes decreased substantially from March 1, 2020, to January 1, 2021 (Figure 2). Measure 4 analyses showed that study nursing homes increased their use of agency staff for all levels of direct care throughout the study period (Figure 3). As a validity check, quantitative results for measures 1 through 4 were compared with the same measures developed for the national sample of 15 436 nursing homes. Changes found for measures 1 to 4 in the study sample of nursing homes were comparable to national changes (Figure 1, Figure 2, and Figure 3).

Thematic results from qualitative analysis were merged and compared with the quantitative results of facility-level staffing data measures. Theme 1 results were evaluated against measures 1 and 2 findings, theme 2 was assessed by measure 4 findings, and theme 3 was evaluated by measure 3 for evidence of agreement.

### Theme 1: Administrators Reported That They Faced Significant Staffing Shortages During the Pandemic

One administrator reflected on the challenge of finding staff throughout the pandemic: “With that many staff members out [due to COVID-19 infection], we had an extreme staffing crisis. There was nobody to help.” (S5N1.3, March 2021). Administrators described an ongoing struggle to maintain safe staff-to-resident ratios, “be in compliance when it comes to staffing” (S6N4.3, June 2021), and provide “good customer service” (S2N4.4, August 2021). Since nursing homes are required to maintain compliance with state and federal regulations around safe staff-to-patient ratios, administrators used compensatory strategies, such as overtime, cross-training, and increasing staff-to-resident ratios to balance regulatory requirements with staffing shortfalls at their facilities. Table 2, theme 1, provides representative quotations.

Using PBJ data to assess staff hours per resident-day per week, our analyses substantiate the qualitative findings indicating that nursing homes faced staffing challenges during the COVID-19 pandemic (theme 1). In support of theme 1, our analyses showed a decrease in total direct care staffing hours for study sample nursing homes throughout the study period (July 14, 2020, to December 16, 2021) (measure 1). As noted in theme 1, administrators used various strategies to compensate for staff shortages and remain in regulatory compliance. Measure 2 analyses of staff hours per resident-day support administrator reports and show an increase of staff hours per resident-day at the outset of the pandemic. The impact of staff shortages extended beyond a facility-wide COVID-19 outbreak with staff hours per resident-day decreasing over time. This was evident at the facilities composing our study sample which, although slightly higher than the national trend, followed the national trend trajectory through December 2021. We continued our analyses through 2022 and found the facilities in our study sample followed national trends but at a higher rate (Figure 1A, B).

### Theme 2: Agency Staff Were Hired to Offset Staffing Shortages

To compensate for staffing shortages, many nursing home administrators brought in agency staff to maintain facility functionality and meet regulatory compliance requirements. As one administrator noted: “We’re still utilizing agency, and all of that to be able to keep the building staffed according to the guidelines” (S6N4.4, September 2021). For some administrators, it was the first time (S2N4.2, February 2021) they had ever needed to rely on agency staff. Hiring agency staff proved problematic during the pandemic as the demand for agency staff soared due to competition with hospitals and other health care settings. As a result, administrators found that agencies could demand high payments for service that was often unreliable. Administrators expressed concerns about the prioritization by agency staff of monetary reward, which negatively impacted existing staff morale and resident care. Table 2, theme 2 presents representative quotations.

Our quantitative analyses using PBJ data support our qualitative findings that nursing home administrators hired agency staff to manage staffing shortages at all levels of patient care (measure 3). Quantitative analyses showed a corresponding change of increasing agency use in both our sample of 40 nursing homes and nationally throughout the study period (July 14, 2020, to December 16, 2021), and continuing in 2022. Additionally, our analyses reflect the increase in agency use by nursing homes for all levels of nursing staff: RNs, LPNs, and CNAs. For our study sample, agency RNs were increasingly used over the course of the study period but at a lower rate than the national average; LPN and CNA agency staff, however, were used at rates higher than national averages by the 40 nursing homes in our study (Figure 2).

### Theme 3: Administrators Reported Being Unable to Increase Their Census Due to Continued Staffing Shortages

A longer-term impact of staff shortages was nursing homes being unable to increase their admissions and census. As one administrator noted: “Yeah, the only restraint on us getting a little bit higher census is staffing issues. We’re running into staffing issues” (S5N2.4, July 2021). For many administrators, low staffing levels impacted their ability to increase their resident census. Thus, curtailing admissions was their only recourse until additional staff could be hired. Table 2, theme 3, presents representative quotations.

Analyses of PBJ data substantiated administrator reports of a decrease in resident census (theme 3). Our analyses (measure 3) found that the number of nursing home residents in our study sample decreased substantially in March 2020 and continued to decrease through January 2021—a trend also found nationally. As nursing home administrators reported, resident census increased through 2022 but, at the time of the study, had not yet reached prepandemic levels (Figure 3).

## Discussion

This study of nursing home administrator perspectives and facility-level staffing data aimed to address a critical gap in understanding how nursing homes met minimum staffing levels and remained operational while experiencing substantial staffing shortages. Using both quantitative and qualitative data, this study may help illuminate crucial ways nursing homes have dealt with the pandemic with 3 important findings.

First, while aggregate staffing and resident census data suggest that resident-to-staff ratios remained stable in the earlier part of the pandemic as a result of the decreasing resident census, our qualitative data provide an important explanatory context not shown by these analyses. Administrators used crisis management compensatory strategies to meet regulatory staffing minimums and maintain operations. As administrators noted, increasing resident-to-staff ratios, hiring agency staff, and reducing resident census enabled them to comply with regulations and continue to care for residents.

Second, although the compensatory strategies administrators used addressed an immediate staffing crisis created by the pandemic, these measures came with a financial cost. Increased staff overtime pay, the high cost of agency staff, and the decreased revenue from new resident admissions has had major financial influences on nursing homes already coping with high operational costs due to the pandemic.^35,36,37^

Third, the stop-gap compensatory mechanisms administrators used to maintain operations have only exacerbated staff burnout. Not only have staff had to manage higher caseloads, they have had the additional burden of supervising and training temporary agency staff unfamiliar with facility protocols. This raises concerns for quality of care at nursing homes as staff burnout and high turnover^38,39^ have been reported to be associated with poor resident outcomes.^40,41,42^

Staffing ratios alone are an incomplete picture of the staffing environment in nursing homes, particularly in the midst of a public health crisis. It took an immense effort with substantial financial and staff costs for nursing homes to maintain minimally adequate staffing ratios and remain operational during the pandemic. The long-term consequences of these compensatory strategies will likely greatly affect the stability of an already strained workforce.^9^

### Limitations

This study has several limitations. First, although our sample size of 40 nursing home administrators in 8 health care markets is robust for qualitative research, our findings may not be generalizable to all US markets and all nursing homes. Our quantitative analyses for facilities participating in this study, however, showed trends that were consistent with national trends. Second, while quantitative analyses continued through September 2022 and show trends continuing, our interviews were conducted between July 14, 2020, and December 16, 2021. Although we were able to capture administrator perspectives close to the beginning of the pandemic, we were not able to explore administrator perspectives into 2022. Third, our interviews focused on nursing home administrator perspectives, which may not represent staff perceptions as they responded to the loss of colleagues and patients, increased workloads, and the influx of agency help.

## Conclusions

Findings from this qualitative mixed-methods study may have implications for future research and policy. The dual approach of quantitative and qualitative analyses provides depth and context to our understanding of complex topics such as staffing and nursing home care. It remains unclear how long the crisis adaptation techniques nursing home administrators used can persist without major effects on staff and resident safety. More mixed-methods research is needed to better understand the long-term outcomes of the COVID-19 pandemic associated with nursing home staffing and how policies and regulations around staffing during a crisis, such as a pandemic, have aided or limited the efficacy of administrator responses to maintain quality care for their residents. Policymakers should consider reviewing current nursing home regulations around staffing and work with nursing home administrators to create policies that more nimbly adjust to crisis management.

## Notes

Supplement 1. **eMethods.** Interview Guides 1 and 4

Supplement 2.
Data Sharing Statement
