# Supplementary material for: Examination of Staffing Shortages at US Nursing Homes During the COVID-19 Pandemic
Source: JAMA Netw Open. 2023 Jul 27;6(7):e2325993. doi: 10.1001/jamanetworkopen.2023.25993 (PMC10375301; doi:10.1001/jamanetworkopen.2023.25993)
Supplement: Supplement 1. — eMethods. Interview Guides 1 and 4 [file jamanetwopen-e2325993-s001.pdf]

Supplementary Online Content

Brazier JF, Geng F, Meehan A, et al. Examination of staffing shortages at US nursing homes during the COVID-19 pandemic. *JAMA Netw Open*. 2023;6(7):e2325993. doi:10.1001/jamanetworkopen.2023.25993

eMethods. Interview Guides 1 and 4

This supplementary material has been provided by the authors to give readers additional information about their work.

© 2023 Brazier JF et al. *JAMA Network Open.*

eMethods. Interview Guides 1 and 4
Interview Guide 1

1. *Introductory and consent to audio record language.*

2. Background

    - Please tell us a bit about being an administrator at this facility.
        - How long have you been admin here? How long have you been working in nursing facilities overall?
        - What is your background (nursing, business, etc.)?

    - Tell me about your facility's residents
        - *Probe*: demographic make-up, variations in age, special populations like mental illness, substance use disorder, other

3. COVID-19 questions

    - What are the most important ways COVID-19 has affected your facility?
        - Residents/patients?
            - *Probe*: visitor restrictions, limitations on new admissions, limits on group activity, other changes to the way that therapy is delivered, census, types of patients/residents, resident/patient fears/concerns, deaths, responding to patient/resident death, responding to patient/resident illness (isolation), symptom screening, testing
        - Family members?
            - *Probe*: visitor restrictions, family fears/concerns
        - Staff?
            - *Probe*: staffing levels, changes in staff workload, hazard pay, symptom screening, staff fears/concerns, responding to staff illness, staff burnout and mental health
        - Other visitors to the facility?
            - *Probe*: contract staff, maintenance/delivery workers

    - You've touched on a number of strategies that you've implemented in your facility- are there any others we haven't yet discussed?
        - *Probe*: care practices, communication with staff, residents, family, etc.
        - How do you anticipate these strategies continuing, even when COVID-19 is no longer a threat?

    - How do you think your facility's physical location has affected COVID-19 in your nursing home? What about the physical building and its layout?

    - How have policies or guidance that the state has put in place impacted your facility and your response to COVID-19?
        - *Probe*: stay at home orders, ban on visitors, waiving of three day minimum hospital stay, requirement that skilled nursing facilities take COVID-19 positive patients (in some states), universal testing of all staff

© 2023 Brazier JF et al. *JAMA Network Open.*

- In many states these policies have been regularly changing- what has that been like?
- How has the state been helpful to your management of COVID-19?
- What help from the state do you still need (information, PPE*, testing, other supplies, staffing, money/resources, liability/legal protection, other)?
- Are there other agencies or organizations that have provided help to your facility? (national guard, city, or other)
- What would be the most important message you'd give to a nursing home administrator who has not [yet] experienced COVID-19?
- What else is important for us to know about how you've weathered COVID-19 at your facility?

4. *Thank you, incentive logistics, and scheduling next interview language.*

*PPE=personal protective equipment

© 2023 Brazier JF et al. *JAMA Network Open.*

Interview Guide 4

1. *Introduction and audio consent language.*

2. *Targeted follow-up questions from previous interview(s).*

3. COVID-19 questions

   Since we talked last, about 3 months ago, in [month]:

   - Have there been any significant changes to your facility's residents?
     - *Probe*: referrals from hospitals (more/less hip/knee replacements, more/less people going right home, acuity, etc.)

   - How are you dealing with conflicting, confusing, or frequently changing policies recently? Are policies and guidelines continuing to be frequently changing or are they more consistent?
     - *Probe*: How are the reporting requirements lately? (less, more, different?) (from Department of Health, CMS*?) What strategies have you used to help with frequent reporting?
     - *Probe*: Reporting frequency, timing and penalties? Where are the data going, and being used for? Accuracy? Anything else?

   - How has COVID-19 affected your facility over the past few months?
     - *Probe*: How is overall morale these days among residents, families and staff?
     - *Probe*: In normal times, families sometimes have difficulties visiting their loved ones in nursing facilities for various reasons. We wonder whether you see families visiting more or less since restrictions have eased. What do they say to you about it? What are your ideas about the increase or decrease in visiting?

     Anything new with staff in the last few months?
     - What methods have you employed or thought of to help with staffing levels?

   - How would you say you, as the administrator, are coping as time goes on?

   - Please give us an update us on vaccinations.
     - *Probe:* new admissions, new staff, making vaccines mandatory in the future?

   - Anything else that we haven't yet discussed?

4. Year in Review: Questions focus on overall, big picture thoughts and ideas

   - A whole year has passed, and we're interested in any major take-aways you've learned. How do you think a facility such as yours could plan or be better able to deal with another massive crisis like COVID-19?
     - *Probe:* things you have done to manage your facility, keep people safe, deal with the media, handle such a big crisis, or anything else.

© 2023 Brazier JF et al. *JAMA Network Open.*

- When you think about the physical structure of your facility, how did it help you manage the pandemic or how did it limit what you could do?
    - *Probe*: number of private rooms, number of wings, number of entrances/exits, number of beds you are budgeted for

- Now that COVID-19 cases are declining in most areas, what priorities might you focus on now in your facility, maybe something you wished you could do, or something new that COVID-19 has made you think of?

- What have been the financial implications of managing COVID-19 at your facility?

- We're interested in how you think the media now reports on nursing homes. How has the media been sympathetic or not over the year?

- How do you think living through this pandemic will impact residents/families/staff in the long term?

- How do you reflect on the industry as a whole now? What might you suggest to your Department of Health or to CMS about ways to improve how nursing homes care for their residents?

- What advice would you give to people interested in going into nursing home administration?

- What else is important for us to know about how you've weathered COVID-19 at your facility?

5. *Thank you, and incentives logistics language*.

*CMS=Centers for Medicare & Medicaid Services

© 2023 Brazier JF et al. *JAMA Network Open.*
