# Supplementary material for: Examination of Staffing Shortages at US Nursing Homes During the COVID-19 Pandemic
Source: JAMA Netw Open. 2023 Jul 27;6(7):e2325993. doi: 10.1001/jamanetworkopen.2023.25993 (PMC10375301; doi:10.1001/jamanetworkopen.2023.25993)
Supplement: Supplement 2. — Data Sharing Statement [file jamanetwopen-e2325993-s002.pdf]

Data Sharing Statement

Brazier. Examination of Staffing Shortages at US Nursing Homes During the COVID-19 Pandemic. *JAMA Netw Open*. Published July 27, 2023.
doi:10.1001/jamanetworkopen.2023.25993

Data
Data available: No
